# Triangle of Care Standards Incorporation and Audit Implementation to Optimise Carer Involvement and Support Services Across Psychiatric Rehabilitation and Acute Wards at Cygnet Churchill Hospital London

**DOI:** 10.1192/bjo.2025.10635

**Published:** 2025-06-20

**Authors:** Angela Misra, Omer Malik, Laura Sheridan, Derek Tracy

**Affiliations:** 1 Cygnet Healthcare, London, United Kingdom.; 2 South London and Maudsley, London, United Kingdom

## Abstract

**Aims:** UK National Institute for Health and Care Excellence Guidelines recognise the importance of effective family and carer involvement in ensuring good patient care and outcomes.

We aim to embed infrastructural changes supporting carer involvement through Cygnet’s Model of Acute and Rehabilitation Care (CMARC), embed rolling audit processes ensuring maintenance of standard adherence across wards and optimise carer support services at Churchill Hospital.

**Methods:** Triangle of Care (TOC) is an alliance between patient, carer and therapeutic staff. The Carers Trust’s TOC partnership (CTTOCP) accreditation was identified as a basis of the initial audit criteria.

Stakeholders were identified and on boarded which included: Cygnet Healthcare Senior Steering Group (CHSSG); Hospital Senior Management Team (SMT); Lambeth Carer’s Hub (local community services) and Carer’s Advocacy Service (CAS). Carer information packs and feedback forms were created by CHSSG and personalised by the Hospital Carer Lead Team (HCLT) for each ward (multidisciplinary clinical and administrative staff) with LCS/CAS sited.

Interventions implemented across 3 audit cycles included 3 areas: formalising communication across stakeholders (shared calendars; audit and carer communications in SMT/Heads Of Department Meetings and Clinical Governance Reports); increasing HCLT personnel (recruitment; Carer Awareness Training and intra hospital promotion); administrative changes (introduction of Carer Communication forms (CCF) clarifying consent status and Carer communication log tables created to improve consistency in record-keeping in ward rounds) and carer engagement initiatives (monthly inter-disciplinary topic-based carer events delivered by HCLT tailored to carer feedback).

Significant changes in results were achieved after the introduction and subsequent iteration of the infographic Carer Involvement Protocol, which aligns with CMARC and Audit criteria in achievable SMART steps. This was disseminated at Stakeholder and HCLT meetings.

**Results:** An audit was carried out in April 2024 with compliance to standards being 87% for rehabilitation and 68% acute wards. Limited carer communication was in place with ad hoc feedback provided. Triangle of care Accreditation was achieved in May 2024. Audit Cycles 2 and 3 in September 2024 and November 2024 both resulted in 100% adherence.

Carer engagement has significantly improved with an increase in attendance overall since conception of monthly events by 28%.

**Conclusion:** There has been significant improvement in the infrastructure of carer services at Churchill Hospital which has relied upon the inter-disciplinary, multi-tiered teamwork and resulted in positive feedback from carers and patient outcomes.

Expert-by-experience led carer events are being introduced in February 2025 with aims to further develop community links and achieve TOC 2 star accreditation.

